# Candesartan Mediated Amelioration of Cisplatin-Induced Testicular Damage Is Associated with Alterations in Expression Patterns of Nephrin and Podocin

**DOI:** 10.1155/2015/273784

**Published:** 2015-10-11

**Authors:** Noritoshi Enatsu, Hideaki Miyake, Koji Chiba, Masato Fujisawa

**Affiliations:** Division of Urology, Kobe University Graduate School of Medicine, 7-5-1 Kusunoki-cho, Chuo-ku, Kobe 650-0017, Japan

## Abstract

Nephrin and podocin are known to be closely related to the pharmacological effects of angiotensin-II receptor blocker (ARB). The objectives of this study were to investigate the role of nephrin and podocin using cisplatin-induced testicular damage and to evaluate the effect of ARB. At first, we evaluated the effects of cisplatin either alone or in combination with ARB candesartan on changes in expression patterns of nephrin and podocin in the rat testes. We then conducted* in vitro* studies to investigate the effects of angiotensin using cultured Sertoli cells, line TM4. As a result, the expression of nephrin and podocin was shown to localize around the basal membrane of seminiferous tubules. Treatment with cisplatin resulted in a marked decrease in the expression of nephrin and podocin and induced a shift of both proteins from linear to granular expression patterns, accompanying the increased apoptotic index in the testes; these changes were partially restored by the additional administration of candesartan.* In vitro* studies with TM4 revealed the angiotensin-II mediated expression changes of nephrin and podocin. These findings suggest that candesartan can prevent cisplatin-induced testicular damage by regulating expression patterns of the nephrin-podocin complex in the testes.

## 1. Introduction

Nephrin and podocin are the well-known proteins, which are connected to each other and play an important role in maintaining the function of the slit diaphragm, a structure likely to be the filtration barrier of the glomerular capillary wall as a highly developed cell-cell junction [1]. Furthermore, several recent studies have reported that the development of proteinuria, not only in congenital nephrotic syndrome but also in acquired renal diseases, is regulated, at least in part, by renal slit diaphragm proteins, including nephrin and podocin [2].

To date, there have been a number of studies demonstrating the involvement of disorders of cell-cell junctions in the development of pathological conditions in the testes [3]. We previously reported the association between impaired spermatogenesis and disorganization of major components of tight junctions in Sertoli cells in both experimental and clinical studies [4, 5]. Collectively, these findings indicate that it might be interesting to investigate whether testicular function is regulated by nephrin and podocin, like some other molecules mediating pathophysiological roles in cell-cell junctions in the testes. In fact, the expression of nephrin and podocin was confirmed in murine testes, and Sertoli cells were suggested to be the testicular origin of these proteins [6, 7]. Furthermore, there have been several studies focusing on angiotensin-II mediated kidney damage through nephrin and podocin. For example, angiotensin-II receptor blocker (ARB) is reported to have antiproteinuric effects which act directly by protecting slit diaphragm molecules, including nephrin and podocin [8, 9], while cisplatin-induced kidney damage is considered to be mediated by angiotensin-II [10, 11]. In fact, the protective effects of ARBs on testis have been previously reported [12–14].

Taken together, in this study, we initially examined the effects of cisplatin-induced damage on expression patterns of nephrin and podocin in rat testes, since platinum analogues like cisplatin are known to have toxic side effects on testicular function and frequently cause azoospermia through the impaired function of Sertoli cells and disruption of blood/testis barrier [15–18]. We then evaluated whether this damage could be restored by the angiotensin-II receptor blocker (ARB), candesartan.

## 2. Materials and Methods

### 2.1. Animals

A total of forty-two 9-week-old male Sprague-Dawley rats weighting 234 ± 23 g were purchased from Oriental Yeast Co. (Tokyo, Japan) and housed in a controlled environment at 22°C on a 12-hour light, 12-hour dark cycle. Animal experiments were approved by Committee of Animal Experiment at Kobe University School of Medicine and conducted in accordance with the National Institutes of Health Guide for the Care and Use of Laboratory Animals [19].

### 2.2. Sertoli Cell Line

The mouse Sertoli cell line TM4 was purchased from the American Type Culture Collection (Manassas, Virginia). Cells were maintained in Dulbecco's Modified Eagle Medium/F12 (DMEM/F12) (Nacalai Tesque, Kyoto, Japan), supplemented with 10% fetal bovine serum. This cell line TM4 is known to form tight junction and is considered suitable for this study [20].

### 2.3. Animal Studies

To examine the* in vivo* effects of treatment with cisplatin (Nichi-Iko Pharmaceutical Co., Toyama, Japan) and/or candesartan, kindly provided by Takeda Pharmaceutical Co. (Osaka, Japan), on expression patterns of testicular nephrin and podocin, a total of 42 rats mentioned above were randomly selected for the following six groups: Group 1 (*n* = 7), intraperitoneal (IP) injection of 2 mL isotonic saline; Group 2 (*n* = 7), IP injection of 2 mL isotonic saline and oral administration of candesartan 5 mg/kg/day; Group 3 (*n* = 7), IP injection of 4 mg/kg cisplatin; Group 4 (*n* = 7), IP injection of 4 mg/kg cisplatin and oral administration of candesartan 5 mg/kg/day; Group 5 (*n* = 7), IP injection of 7 mg/kg cisplatin; and Group 6 (*n* = 7), IP injection of 7 mg/kg cisplatin and oral administration of candesartan 5 mg/kg/day. After randomization, either candesartan at a dose of 5 mg/kg or water was orally administered once daily for 24 days, and on day 3, either cisplatin at a dose of 4 or 7 mg/kg or isotonic saline was given by IP injection. The dose and duration after treatment of cisplatin and candesartan were determined based on those reported in the previous study and our preliminary experiments [21, 22].

Three weeks after treatment, rats were sacrificed, and testes and epididymides were removed. Testicular weight measurement and epididymal semen analysis were performed on both sides, and mean values were used for the calculation. After semen analysis, testes were bisected, and one half was snap-frozen immediately and stored at −80°C until assessed, and the remainder was fixed in Bouin solution for histopathological examination.

### 2.4. Sperm Analysis

The epididymal sperm concentration was determined by using the modified method described by Okada et al. [23]. Briefly, the epididymis was finely minced by anatomical scissors within 1 mL of modified Whitten's medium (15 mM Hepes-sodium salt, 1.2 mM MgCl_2_, 100 mM NaCl, 4.7 mM KCl, 1 mM pyruvic acid, and 4.8 mM lactic acid) in a dish and then allowed to incubate at room temperature for 1 hour to provide the migration of all spermatozoa from epididymis. Sperm analysis, including the number and motility, was performed using Makler counting chamber (Irvine Scientific, CA, USA). A total of 20 fields were analyzed for each sperm sample. In order to detect the spermatozoa with abnormal morphologies, the Diff-Quick kit (Medion Diagnostics International, FL, USA) was used to stain the smears of semen samples. Diff-Quick fixing and staining times were 5 minutes and the slides were washed in distilled water to eliminate excess staining, air-dried, and covered with a coverslip. At least 100 sperm per slide were counted to determine the percentage of spermatozoa with abnormal morphologies.

### 2.5. Treatment of TM4 Cells

To examine the changes in expression patterns of nephrin and podocin in the cultured TM4 cells, the cells were treated with 2 × 10^−8^ mL/L angiotensin-II (A9525, Sigma-Aldrich, Tokyo, Japan) for 1 hour in the presence or absence of 2.5 × 10^−6^ mol/L candesartan. Following treatment, cells were harvested and used for subsequent experiments. Angiotensin-II is considered suitable for our experiment, to investigate the involvement of angiotensin system through nephrin and podocin. That is, angiotensin receptor is located in Sertoli cell, while there is no production of angiotensin-II from Sertoli cell [24, 25].

### 2.6. Real-Time RT-PCR

Total ribonucleic acid (RNA) was extracted from the rat testes and cultured TM4 cells using TRIzol reagent (Life Technologies Japan, Tokyo, Japan). RT reactions were carried out using a GeneAmp RNA PCR Kit (Applied Biosystems, Foster City, California) according to the manufacturer's instructions. To quantitatively determine the expression levels of nephrin, podocin, and GAPDH mRNAs in each sample, real-time PCR analysis using a standard curve method with SYBR Green I (Takara Bio, Tokyo, Japan) was then conducted as previously described [26]. The sequence-specific primers, synthesized by Operon Biotechnology Inc. (Tokyo, Japan) based on previous studies, are presented in Table 1 [27, 28]. Data were normalized with glyceraldehyde 3-phosphate dehydrogenase (GAPDH) values and expressed as arbitrary units relative to samples from control, regarded as “1.”

### 2.7. Western Blot Analysis

Samples containing equal amounts of protein (20 *μ*g) from lysates of the rat testes or cultured TM4 cells were subjected to SDS-polyacrylamide gel electrophoresis and transferred to a nitrocellulose filter. The filter was blocked in PBS containing 5% nonfat milk powder at 4°C overnight and then incubated for 1 hour with antibodies against nephrin (1 : 200, sc-32532, Santa Cruz Biotechnology, Santa Cruz, California), podocin (1 : 200, P0372, Sigma-Aldrich, Tokyo, Japan), and *β*-actin (1 : 1000, A1978, Sigma-Aldrich). The filters were then incubated for 30 minutes with horseradish peroxide-conjugated secondary antibodies (1 : 2000, sc-2004, sc-2020 Santa Cruz Biotechnology), and specific proteins were detected using an enhanced chemiluminescence Western blot analysis system (RPN2106, GE Healthcare, Tokyo, Japan). The relative optical densities of the bands were quantified using Image J Program [29], and data were expressed as corresponding values of the ratio relative to the result from control group.

### 2.8. Immunohistochemical Studies

Immunofluorescence staining was used to investigate the expression of nephrin and podocin. For frozen sections, antibodies against nephrin and podocin as described above were added in dilution of 1 : 200 and 1 : 400, respectively, and then incubated for 2 hours. After staining with the secondary antibodies goat anti-rabbit IgG-TR (1 : 500, sc-2780, Santa Cruz Biotechnology) and donkey anti-goat IgG-FITC (1 : 500, sc-2024, Santa Cruz Biotechnology), the sections were mounted and the staining outcomes were evaluated by inverted fluorescence phase-contrast microscopy (Keyence BZ-9000, Osaka, Japan). For* in vitro* studies, cultured TM4 cells were seeded on coverslips and fixed in 4% paraformaldehyde and were stained with primary and secondary antibodies, the same as* in vivo* study, and counterstaining with 4′,6-diamidino-2-phenylindole (DAPI) was additionally performed.

### 2.9. TUNEL Staining

Apoptotic features in the rat testes were evaluated by Terminal Deoxynucleotidyl Transferase-mediated dUTP Nick End-labelling (TUNEL) assay using an* in situ* cell death detection kit (11684795910, Roche Applied Science, Penzberg, Germany) according to the manufacturer's instructions. Quantitative analysis was performed by counting the number of apoptotic cells and total germ cells in 100 randomly selected tubules in one slide per animal.

### 2.10. Immunofluorescence Staining of Carbonylated Protein

Carbonylated protein is known as a marker of oxidative stress, since protein carbonylation has been identified as a potential mechanism underlying mitochondrial dysfunction which is linked to oxidative stress [30, 31]. The expression of carbonylated protein in the rat testes was evaluated using a protein carbonyls immunohistochemical staining kit (Cosmo Bio, Tokyo, Japan). In accordance with the manufacturer's protocol, methacarn-fixed paraffin-embedded sections were incubated with anti-dinitrophenylhydrazine (DNP) antibody, followed by incubation with fluorescein isothiocyanate (FITC) conjugated secondary antibody for 1 hour at room temperature in the dark. After the labelling procedure was completed, the coverslips were mounted onto glass slides using a mounting medium containing 4′,6-diamidino-2-phenylindole.

### 2.11. Superoxide Dismutase (SOD) Assay

To quantify the production of superoxide in the rat testes, Superoxide Dismutase (SOD) activity was measured using an SOD Assay Kit-WST (DOJINDO, Kumamoto, Japan) based on the manufacturer's protocol. In this assay, homogenized tissue samples were centrifuged and the supernatant was processed for the measurement of SOD activity as previously described [32]. One unit of SOD activity was defined as the amount of enzyme that inhibits 50% of the WST-1 formazan per minute.

### 2.12. Immunoprecipitation

Immunoprecipitation analysis was performed as previously described procedure [33]. Homogenized samples were precleared by incubation with protein G-plus-agarose beads (sc-2003, Santa Cruz Biotechnology) at 4°C for 1 hour and then incubated with primary antibodies as described above. Agarose beads were washed with lysis buffer and suspended in SDS/PAGE sample buffer. After SDS-polyacrylamide gel electrophoresis, samples were analyzed by Western blotting with corresponding antibodies.

### 2.13. Statistical Analysis

The significance of differences was evaluated by the Mann-Whitney *U* test for unpaired observations. Values were expressed as the mean ± standard deviation, and *P* values < 0.05 were considered significant.

## 3. Results

**Figure 1:**
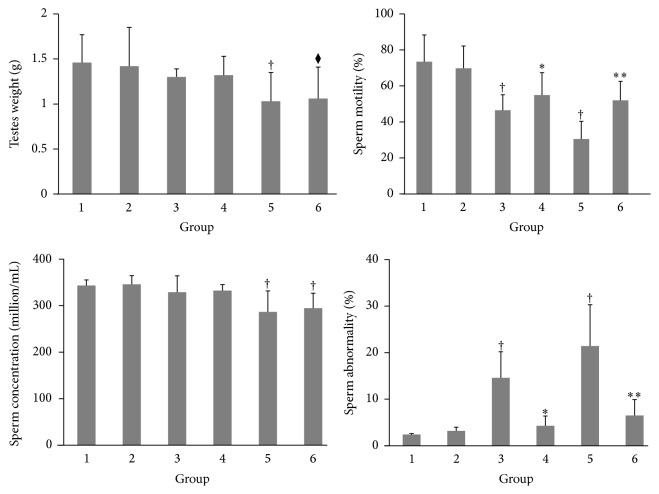
Weights of testes, epididymal sperm concentrations, and percentages of sperm motilities and abnormalities following treatment with cisplatin and/or candesartan. Group 1 (*n* = 7), intraperitoneal (IP) injection of 2 mL isotonic saline; Group 2 (*n* = 7), IP injection of 2 mL isotonic saline and oral administration of candesartan 5 mg/kg/day; Group 3 (*n* = 7), IP injection of 4 mg/kg cisplatin; Group 4 (*n* = 7), IP injection of 4 mg/kg cisplatin and oral administration of candesartan 5 mg/kg/day; Group 5 (*n* = 7), IP injection of 7 mg/kg cisplatin; and Group 6 (*n* = 7), IP injection of 7 mg/kg cisplatin and oral administration of candesartan 5 mg/kg/day. Each column represents the mean value with standard deviation. ^†^, ^*∗*^, and ^*∗∗*^ differ from Groups 1, 3, and 5, respectively (*P* < 0.05).

The changes in testicular weight and sperm analysis following treatment with cisplatin and/or candesartan are summarized in Figure 1. Cisplatin caused the dose-dependent testicular damage; administration of high-dose (7 mg) cisplatin caused a significant decrease in testicular weights, while low-dose (4 mg) cisplatin did not affect the testicular weight. These changes were not affected by candesartan treatment. Sperm analysis revealed that sperm concentration and motility were significantly decreased by cisplatin administration, and increased proportion of abnormal sperm was observed after cisplatin administration. Coadministration of candesartan significantly recovered the motility rate and sperm abnormality rate. Sperm concentration was slightly recovered by candesartan, but the difference was not statistically significant.

**Figure 2:**
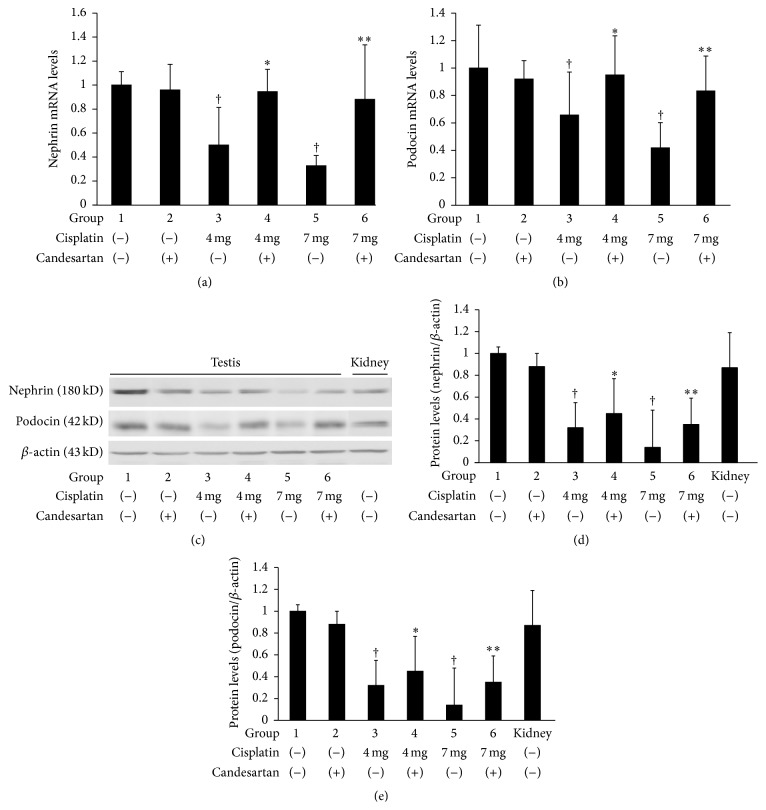
Changes in expression levels of nephrin and podocin in the testes of rats following treatment with cisplatin and/or candesartan. (a) Total RNA was extracted from the testes and analyzed for nephrin mRNA levels by real-time RT-PCR. Each column represents the mean value with standard deviation. ^†^, ^*∗*^, and ^*∗∗*^ differ from Group 1, Group 3, and Group 5, respectively (*P* < 0.05). (b) Total RNA was extracted from the testes and analyzed for podocin mRNA levels by real-time RT-PCR. Each column represents the mean value with standard deviation. ^†^, ^*∗*^, and ^*∗∗*^ are represented as in (a). (c) Protein was extracted from the testes, and expression levels of nephrin, podocin, and *β*-actin were analyzed by Western blotting. (d) Nephrin and *β*-actin signal intensity in (c) were quantified, and the ratio of nephrin over *β*-actin (d) and that of podocin over *β*-actin (e) were presented in histogram format. ^†^, ^*∗*^, and ^*∗∗*^ are represented as in (a).

We then assessed the changes in the expression levels of nephrin and podocin in the rat testes following treatment with cisplatin and/or candesartan. The expression of both nephrin and podocin mRNAs was detectable in the rat testes, and these levels were significantly decreased by treatment with cisplatin (Figures 2(a) and 2(b)); however, these changes were partially, but significantly, restored by combined administration with candesartan. Changes in the protein expression levels of both nephrin and podocin after treatment with cisplatin and/or candesartan were similar to those in the nephrin and podocin mRNAs (Figures 2(c)–2(e)).

**Figure 3:**
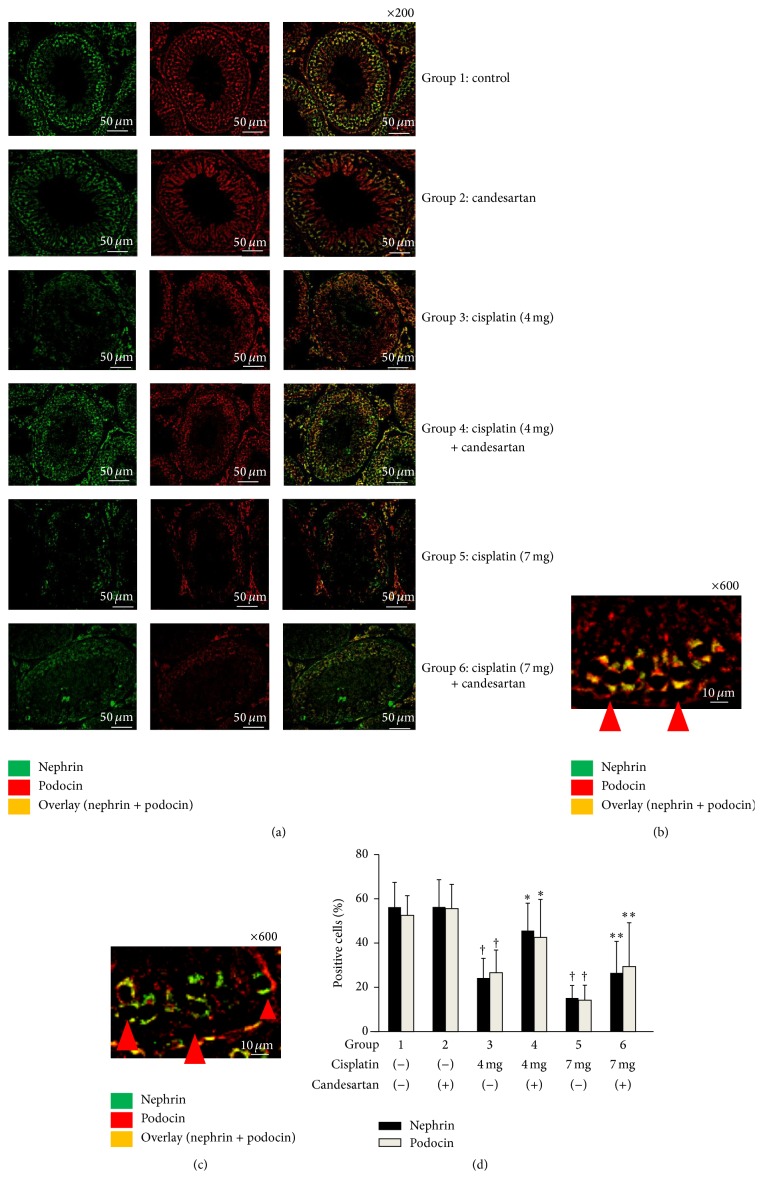
Immunofluorescence double staining for nephrin and podocin in the testes of rats following treatment with cisplatin and/or candesartan. Nephrin was stained in green with FITC, podocin was stained in red with Texas Red, and the nuclei were stained in blue with DAPI. The overlaps of nephrin and podocin are indicated in orange. (a) Representative findings of immunofluorescence double staining and changes in each group. (b) High magnification images of control testis. Arrows indicate that nephrin and podocin are coexpressed in linear formation near basal membrane of seminiferous tubules. (c) High magnification images of testis following treatment with cisplatin. Arrows indicate that, after cisplatin (7 mg) administration, the expression patterns of nephrin and podocin were dissociated and in discrete granular structure. (d) Quantitative analysis of immunofluorescence positive cells for nephrin and podocin. ^†^, ^*∗*^, and ^*∗∗*^ differ from Group 1, Group 3, and Group 5, respectively (*P* < 0.05).

Expression patterns of nephrin and podocin in the rat testes following treatment with cisplatin and/or candesartan are presented in Figure 3. Dual-labeling immunofluorescence staining revealed colocalization of nephrin and podocin near the basal membrane of seminiferous tubules. Moreover, the administration of cisplatin markedly reduced the staining intensities of both nephrin and podocin and resulted in a shift of both proteins from linear to granular expression patterns, accompanying the dissociation of these two molecules. These changes induced by cisplatin were partially restored by combined treatment with candesartan.

**Figure 4:**
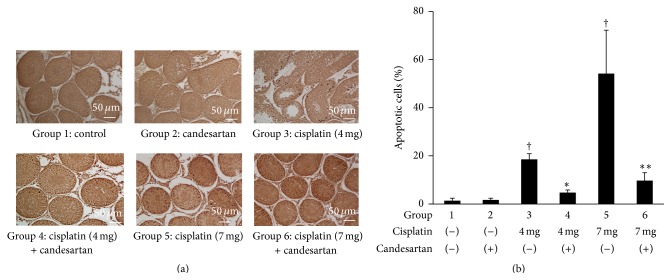
Apoptotic features in the testes of rats following treatment with cisplatin and/or candesartan. (a) Representative findings of TUNEL staining in rat testes. (b) The numbers of TUNEL-positive cells and total germ cells in 100 randomly selected tubules in one slide per animal were counted. Apoptotic index, defined as the percentage of TUNEL-positive cells, was then calculated for each slide. Each column represents the mean value with standard deviation. ^†^, ^*∗*^, and ^*∗∗*^ differ from Group 1, Group 3, and Group 5, respectively (*P* < 0.05).

**Figure 5:**
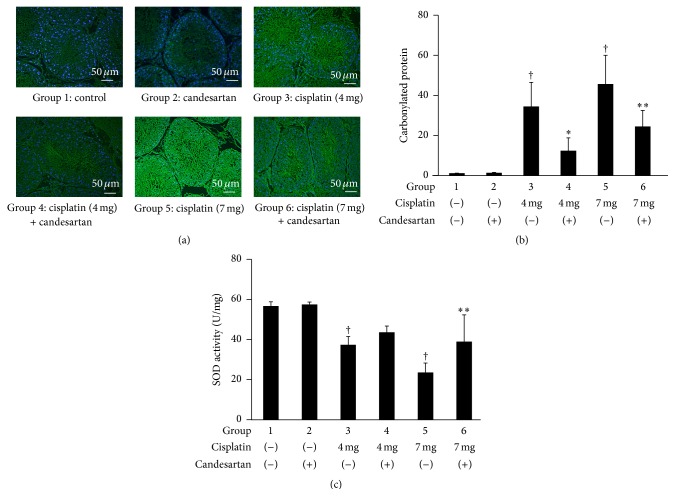
Oxidative stress status in the testes from rats following treatment with cisplatin and/or candesartan. (a) Representative findings on immunofluorescent staining of carbonylated protein in the rat testes. (b) Carbonylated protein signal intensities in (a) were quantified by Image J software and the ratio of each group over Group 1 was presented in histogram format. (c) Homogenized testicular specimens were prepared and analyzed for SOD activity. Each column represents the mean value with standard deviation. ^†^, ^*∗*^, and ^*∗∗*^ differ from Group 1, Group 3, and Group 5, respectively (*P* < 0.05).

The effects of treatment with cisplatin and/or candesartan on apoptotic features in the rat testes were subsequently investigated. As shown in Figure 4, a marked increase in the number of TUNEL-positive cells was observed in the group treated with cisplatin in a dose-dependent manner, while the number of TUNEL-positive cells was significantly reduced in the group receiving candesartan in addition to cisplatin. Furthermore, the assessment of oxidative stress revealed that treatment with cisplatin induced a marked increase in the expression of carbonylated protein in the rat testes compared with the baseline expression in the control group without cisplatin treatment, and coadministration of candesartan partially inhibited the cisplatin-induced upregulation of this protein (Figures 5(a) and 5(b)). Similarly, a quantitative assessment of SOD activity in the rat testes showed a cisplatin-induced decrease in this activity and its partial restoration by candesartan (Figure 5(c)).

**Figure 6:**
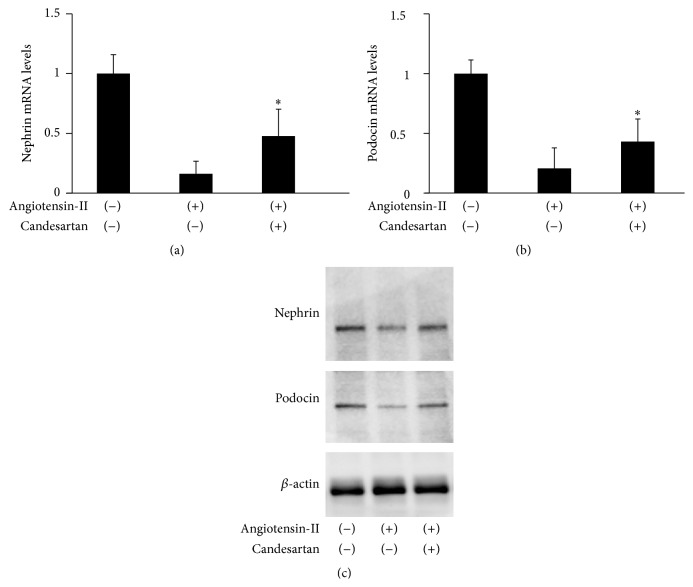
Changes in expression levels of nephrin and podocin in cultured mouse Sertoli cells, line TM4, following treatment with angiotensin-II and/or candesartan. (a) Total RNA was extracted from TM4 cells and analyzed for nephrin mRNA levels by real-time RT-PCR. Each column represents the mean value with standard deviation. ^*∗*^ differs from TM4 cells treated with cisplatin alone (*P* < 0.05). (b) Total RNA was extracted from the testes and analyzed for podocin mRNA levels by real-time RT-PCR. Each column represents the mean value with standard deviation. ^*∗*^ differs from TM4 cells treated with angiotensin-II alone (*P* < 0.05). (c) Protein was extracted from TM4 cells and expression levels of nephrin, podocin, and *β*-actin were analyzed by Western blotting.

**Figure 7:**
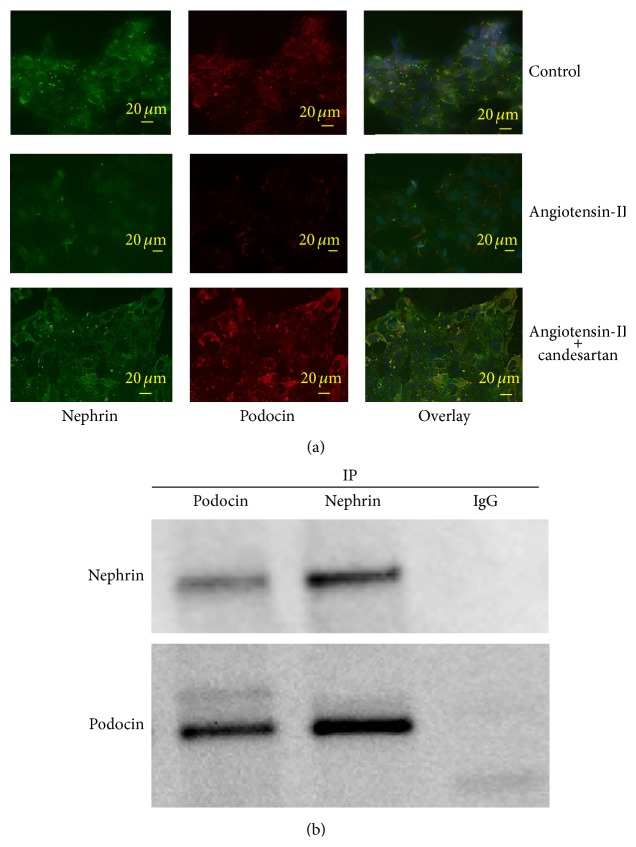
(a) Immunofluorescence double staining for nephrin and podocin in cultured mouse Sertoli cells, line TM4, following treatment with angiotensin-II and/or candesartan. Nephrin was stained in green with FITC, podocin was stained in red with Texas Red, and the nuclei were stained in blue with DAPI. The overlaps of nephrin and podocin are indicated in orange. Nephrin and podocin are mainly located at junctions between cells, and administration of angiotensin-II induced redistribution of nephrin and podocin. These changes were partially restored by additional treatment with candesartan. (b) Coimmunoprecipitation (IP) assays of nephrin and podocin in cultured mouse Sertoli cells, line TM4. Whole-cell lysates prepared from TM4 cells were immunoprecipitated with antipodocin or nephrin antibody and blotted with antinephrin or podocin antibody, respectively.

To confirm the findings of* in vivo* experiments,* in vitro* studies using a cultured mouse Sertoli cell line, TM4, were conducted. In TM4 cells as well, nephrin and podocin were definitely expressed at both mRNA and protein levels, and treatment with angiotensin-II induced a reduction of their expression levels, which could be partially recovered by additional treatment with candesartan (Figure 6). As shown in Figure 7(a), immunofluorescence staining revealed that the expression of nephrin and podocin was mainly localized at junctions between TM4 cells cultured in standard medium; however, exposure of TM4 cells to angiotensin-II induced redistribution of these proteins, resulting in the emergence of diffused localization patterns. These changes in expression patterns of both proteins could be restored by combined treatment with candesartan.

To examine whether nephrin specifically binds to podocin in TM4 cells, immunoprecipitation assay was performed. As shown in Figure 7(b), nephrin was coimmunoprecipitated with podocin, while podocin was also coimmunoprecipitated with nephrin.

## 4. Discussion

During the last two decades, several molecules have been identified to be associated with the structure of the slit diaphragm in the kidney podocyte, the primary filtration barrier responsible for macromolecule proteins. The initially reported molecule consisting of the slit diaphragm was ZO-1, originally identified as a component of the tight junction [34]. Nephrin and podocin, also well characterized components of the slit diaphragm, were shown to bind to each other, and several studies suggested that abnormal or insufficient signaling via the nephrin-podocin complex is likely to result in the development of proteinuria [8, 27, 35]. Recently, Relle et al. reported that the expression of podocin was detected in the testes and that confocal laser microscopy revealed colocalization of podocin with filamentous actin within Sertoli cells, suggesting a possible role of podocin in the blood-testis barrier [6]. Liu et al. reported the testicular expression of nephrin as well, which was colocalized with ZO-1 [7]. In this study, we revealed the colocalization of nephrin and podocin near the basal membrane of seminiferous tubules. These localization patterns were similar to those of tight junction protein, such as F-actin, *β*-catenin, and N-cadherin [6, 36]. Collectively, these findings indicate that nephrin and podocin may be involved in the function mediating the barrier system in cell-cell junction in the testis.

In this study, in order to investigate the relationship between testicular damage and expression patterns of nephrin and podocin in the testes, we treated rats with cisplatin, which has been shown to have a potential toxic effect on the testes through the impairment of functions associated with cell-cell adhesion [17, 37]. We initially confirmed a significant decrease in sperm concentration and motility as well as increased proportion of abnormal sperm in rats treated with cisplatin. Furthermore, cisplatin treatment induced marked inhibitory effects on the expression of testicular nephrin and podocin. Immunohistochemical analyses also showed that nephrin and podocin were colocalized near the basal membrane of seminiferous tubules without cisplatin treatment; however, the dissociated expression of these proteins, accompanying the changes in granular expression patterns, was observed following the administration of cisplatin. These findings suggest the possible role of nephrin and podocin in cisplatin-induced damage through the disruption of the barrier function in the testes.

It is of interest to investigate whether cisplatin-induced testicular damage can be alleviated by an antiproteinuric agent. It has been well recognized that ARBs help reduce renal tissue damage and proteinuria in some renal diseases through specific blockade of angiotensin-II type 1 receptor (AT1R), independently of its function to lower blood pressure [32, 33]. Furthermore, several recent studies have demonstrated that AT1R-mediated action directly protects slit diaphragm molecules, including nephrin and podocin, from stimuli-inducing renal injury [8, 9, 38, 39]. To evaluate the regulatory mechanism of nephrin and podocin by angiotensin-II in addition to these findings, we assessed the effects of candesartan, one of the most prevalent ARBs, on the damage in rat testes following cisplatin treatment. Our results showed that the administration of candesartan resulted in the improvement of cisplatin-induced toxic events in the rat testis, including decreased sperm motility, and increased proportion of abnormal sperm. In addition, the reduced expression of nephrin and podocin and their dissociated expression patterns accompanied by apoptosis were partially restored by candesartan administration. Other than the present findings, there have been several studies revealing the involvement of an angiotensin-mediated system in the maintenance of testicular function [12–14, 40, 41]. For example, Shiraishi et al. reported that AT1R-dependent fibrosis in rat testes was observed after vasectomy and that impaired spermatogenesis caused by interstitial fibrosis was partially abrogated by ARB administration [14]. Similarly, Kushwaha and Jena reported that testicular toxicity induced by nicotine in streptozotocin-induced diabetic rat was attenuated by ARB treatment [13]. Taken together, these findings indicate that ARB may have protective effects from testicular damage induced by several types of toxic stimuli to the testes.

**Figure 8:**
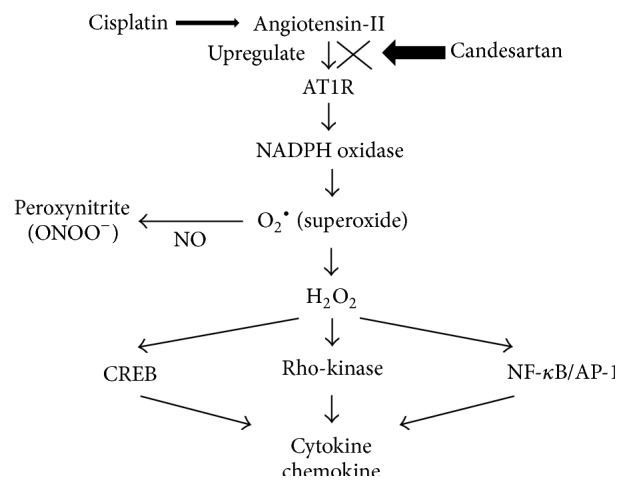
Signaling pathway of angiotensin-II type 1 leading to superoxide production. AT1R: angiotensin-II type 1 receptor, NADPH: nicotinamide adenine dinucleotide, CREB: cAMP response element binding protein, and NO: nitric oxide.

Another point of interest is the mechanism mediating the protective effects on cisplatin-induced testicular damage by candesartan. To date, a number of previous studies have reported the protective effects of ARB on testicular damage. Some of these reports clarified the antioxidative activity induced by ARB as one of the main mechanisms underlying its protective effect on testicular damage [13, 14]. In fact, there have been several studies showing the induction of reactive oxygen species (ROS) through AT1R as well as the antioxidant activity of ARB in various types of cells [42] (Figure 8). Griendling et al. reported that angiotensin-II specifically activates enzyme systems that promote superoxide generation in cultured vascular smooth muscle cells [43]. Similarly, Okui et al. showed that cisplatin increased serum levels of angiotensin-II and aldosterone, which synergistically prolonged the accumulation of cisplatin in renal tissues [10]. In the testes as well, ROS has been shown to produce detrimental effects on the maintenance of their physiological functions [44]. Furthermore, antioxidants have been regarded as having protective effects on testicular damage [45–47]. For example, Salem et al. also reported the attenuation of cisplatin-induced testicular toxicity by antioxidant supplementation with selenium and lycopene [48]. Similarly to these studies, we also confirmed the restoring effect of candesartan on increased oxidative stress induced by cisplatin in rat testes. Considering these findings, candesartan may alleviate cisplatin-induced testicular damage through the inhibition of oxidant activity. However, in addition to these findings, we have to mention the toxic effects of cisplatin on cells in the adluminal compartment, which are normally protected by the blood/testis barrier, indicating that cisplatin and candesartan might modulate the functions of blood/testis barrier. In particular, ARB mediated protective effects on cell-cell junction in the testes, including nephrin and podocin. In this study, we confirmed that the dissociated nephrin-podocin complex was recovered by ARB administration, indicating that ARB could have protective effects among blood/testis barrier architecture.

In order to confirm and reinforce the findings obtained from* in vivo* studies, we also performed several* in vitro* experiments using a mouse Sertoli cell line, TM4. As for the changes in expression patterns of nephrin and podocin in TM4 cells following treatment with angiotensin-II and/or candesartan, we achieved findings that angiotensin-II directly acts on Sertoli cells and affects the expression of nephrin and podocin. Furthermore, the immunoprecipitation assay demonstrated binding between nephrin and podocin in TM4 cells. To our knowledge, this is the first study providing evidence concerning the binding of these proteins in cells other than those derived from the kidney. Although additional studies will be necessary to address the functional significance of the binding between testicular nephrin and podocin, these findings strongly suggest a direct interaction between these proteins in the testes like that in the renal slit diaphragm.

Here, we would like to emphasize several limitations of this study. Firstly, treatment of cisplatin was selected to induce damage in the testes; therefore, it is impossible to apply the findings of this study to all types of testicular injury. Secondly, we did not directly address whether testicular nephrin and podocin colocalized in tight junction in seminiferous tubules and regulate the physiological testicular functions or blood-testis barrier. Thus, it would be required to perform further study, such as that using immunoelectron microscopy and that assessing blood-testis barrier permeability. Thirdly, the significance of the use of candesartan together with chemotherapeutic agents from a clinical viewpoint should be evaluated. Finally, expression patterns of the nephrin-podocin complex in clinical testicular specimens from infertile men should be investigated to assess the role of these proteins in spermatogenesis.

## 5. Conclusion

Candesartan can prevent cisplatin-induced testicular damage by regulating expression patterns of the nephrin-podocin complex, which was shown to be localized near the basal membrane of seminiferous tubules in the testes. These findings suggest the possible involvement of angiotensin-II mediated action on these proteins in executing physiological functions as a component of cell-cell junction in the testes.

## Figures and Tables

**Table 1:** The species-specific primers of podocin, nephrin and GAPDH.

	Sense primer	Antisense primer
Rat		
Podocin	CCTGTGAGTGGCTTCTTGTCCTC	GGAGACGCTTCATAGTGGTTTGCA
Nephrin	CTGACTGGGCTGAAGCCTTCT	AAGAGCACAGGCAGCAGGGG
GAPDH	CTCTACCCACGCCAAGTTCAA	GGATGACCTTGCCCACAGC
Mouse		
Podocin	TGAGGATGGCGGCTGAGAT	GGTTTGGAGGAACTTGGGT
Nephrin	CCCAACACTGGAAGAGGTGT	CTGGTCGTAGATTCCCCTTG
GAPDH	CTCATGACCACAGTCCATGC	CACATTGGGGGTAGGAACAC

## Abbreviations

ARB: Angiotensin-II receptor blocker
AT1R: Angiotensin type 1 receptor
DAPI: 4′,6-Diamidino-2-phenylindole
DMEM/F12: Dulbecco's Modified Eagle Medium/F12
DNP: Dinitrophenylhydrazine
FITC: Fluorescein isothiocyanate
GAPDH: Glyceraldehyde 3-phosphate dehydrogenase
IP: Intraperitoneal
RNA: Ribonucleic acid
ROS: Reactive oxygen species
RT-PCR: Reverse-transcriptase polymerase chain reaction
SDS: Sodium dodecyl sulfate
SOD: Superoxide Dismutase
TUNEL: Terminal Deoxynucleotidyl Transferase-mediated dUTP Nick End-labelling.
